# Artificial Intelligence-Based Diagnosis of Oral Lichen Planus Using Deep Convolutional Neural Networks

**DOI:** 10.1055/s-0042-1760300

**Published:** 2023-01-20

**Authors:** Paniti Achararit, Chawan Manaspon, Chavin Jongwannasiri, Ekarat Phattarataratip, Thanaphum Osathanon, Kraisorn Sappayatosok

**Affiliations:** 1 Princess Srisavangavadhana College of Medicine, Chulabhorn Royal Academy, Bangkok, Thailand; 2 Biomedical Engineering Institute, Chiang Mai University, Chiang Mai, Thailand; 3 Department of Oral Pathology, Faculty of Dentistry, Chulalongkorn University, Bangkok, Thailand; 4 Dental Stem Cell Biology Research Unit, Department of Anatomy, Faculty of Dentistry, Chulalongkorn University, Bangkok, Thailand; 5 College of Dental Medicine, Rangsit University, PathumThani, Thailand

**Keywords:** oral lichen planus, convolution neural network, AI-based diagnosis, oral lesions, artificial intelligence

## Abstract

**Objective** The aim of this study was to employ artificial intelligence (AI) via convolutional neural network (CNN) for the separation of oral lichen planus (OLP) and non-OLP in biopsy-proven clinical cases of OLP and non-OLP.

**Materials and Methods** Data comprised of clinical photographs of 609 OLP and 480 non-OLP which diagnosis has been confirmed histopathologically. Fifty-five photographs from the OLP and non-OLP groups were randomly selected for use as the test dataset, while the remaining were used as training and validation datasets. Data augmentation was performed on the training dataset to increase the number and variation of photographs. Performance metrics for the CNN model performance included accuracy, positive predictive value, negative predictive value, sensitivity, specificity, and F1-score. Gradient-weighted class activation mapping was also used to visualize the important regions associated with discriminative clinical features on which the model relies.

**Results** All the selected CNN models were able to diagnose OLP and non-OLP lesions using photographs. The performance of the Xception model was significantly higher than that of the other models in terms of overall accuracy and F1-score.

**Conclusions** Our demonstration shows that CNN models can achieve an accuracy of 82 to 88%. Xception model performed the best in terms of both accuracy and F1-score.

## Introduction

Oral lichen planus (OLP) is a chronic inflammatory autoimmune disease. There are several clinical presentations of OLP, including the classic white reticular pattern, erosive, atrophic, plaque, and bullous lesions. According to the modified World Health Organization (WHO) diagnostic criteria of OLP, 2003, the erosive, atrophic, bullous, and plaque-like lesions are the only accepted subtype in the presence of reticular lesions. 1 The clinical criteria of OLP diagnosis in modified WHO 2003 include the presence of bilateral, more or less symmetrical lesions and the presence of lace-like network of slightly raised gray-white line. 1 The histopathological criteria of OLP in modified WHO 2003 include well-defined lymphocytic band zone confined in the superficial connective tissue layer, basal cell layer liquefactive degeneration, and absence of epithelial dysplasia. 1 Another clinical spectrum of lesions resembling OLP, but with known causes, includes oral lichenoid lesions (OLL). Clinical and histopathological features cannot differentiate between these two types of lesions. World Workshop in Oral Medicine IV in 2006 classified OLP and OLL into four distinctive groups: classic OLP, oral lichenoid drug reactions, oral lichenoid contact lesions, and oral lichenoid graft-versus host disease. 2 Approximately 50% of patients with skin lesions also manifest oral mucosal lesions, and 25% of patients with OLP present with only oral lesions. 3 OLP and OLL may involve any part of the oral mucosa, predominantly the buccal mucosa and gingiva and can also present as desquamative gingivitis.

Currently, both OLP and OLL are classified as oral potentially malignant disorders (OPMD). 4 The malignant transformation rate of OLP is approximately 1.37% and is slightly higher for OLL at 2.43%. 5 Erosive type, female sex, tongue lesions, smoking, alcoholism, and hepatitis C infection are risk factors for malignant transformation of OLP and OLL. 5 6 The clinical differential diagnosis of OLP and OLL includes frictional keratosis, pseudomembranous candidiasis, erythematous candidiasis, leukoplakia, lupus erythematosus, pemphigus vulgaris, mucous membrane pemphigoid, and chronic ulcerative stomatitis.

Artificial intelligence (AI) is a new technology that has contributed to several medical and dental fields. AI, including machine learning (ML) 7 and deep learning (DL), has shown promising results and has been proven to be an effective method for the diagnosis of oral diseases, such as dental caries 8 and odontogenic lesions. 9

In DL, AI can imitate the human brain using the neural network structure of the deep layer. The machine can repeatedly learn and gain knowledge from the trained data. The principles of DL involve the standard DL model, convolutional neural network (CNN) using object recognition, and classification of images from the data put in the system. For example, the difference in the gradient of radiodensity, either radiolucency or radiopacity, can be extracted and analyzed using AI. AI takes the differences as an input and differentiates between the two radiodensities by repeated learning. Moreover, the shape, contour, color, and pattern of the objects can also be analyzed using AI.

In their study, Ariji et al showed that the sensitivity for detecting metastatic and nonmetastatic lymph nodes in computed tomography image reached 90 and 80%, respectively. 10 Oral cancer detection using CNN from photographic images yielded a sensitivity of 94.9 and specificity of 88.7%. 11 The application of AI can also be combined with other modalities, such as fluorescent confocal microscopy, which yields a sensitivity of 96%. 12 Furthermore, histopathological diagnosis of oral squamous cell carcinoma using CNN has a sensitivity of 98% and specificity of 92%. 13 The application of CNN using panoramic radiographs has also shown significant results, including diagnosis of radiolucent lesions, 14 mesiodens, 15 taurodontism, 16 cystic lesions, 17 or even fractures. 18

Clinical diagnosis of OLP and how to separate it from other white, white-red, and red lesions maybe difficult for general practitioner. And there are no studies clarifying performance of AI in OLP diagnosis before. Thus, in this research, we aimed to employ AI via CNN for the differentiation of OLP and non-OLP in biopsy-proven clinical cases of OLP and non-OLP.

## Materials and Methods

### Data Preparation

Clinical photographs of OLP and non-OLP lesions from the archive of the College of Dental Medicine, Rangsit University, were collected for this study. The convenience sampling method was used to create the dataset which the total of clinical photographs in the archive were 1089. The photographs were then categorized into two classes: 609 for OLP and 480 for non-OLP lesions. The final diagnoses of both groups of some lesions were confirmed histopathologically. Non-OLP photographs include lesion that should be included in the differential diagnoses of OLP such as hyperkeratosis, oral epithelial dysplasia, carcinoma in situ, recurrent aphthous ulcer, traumatic ulcer, pemphigus vulgaris, mucous membrane pemphigoid, lupus erythematosus, and erythematous candidiasis.

The photographs were cropped to remove unnecessary areas (e.g., medical instruments, hands, teeth) so that the CNN could focus only on the OLP and non-OLP lesions. The edited photographs were saved in 8-bit JPEG format.

The photographs were then separated into two datasets, and 55 photographs from the OLP and non-OLP groups were randomly selected for use as the test dataset. The remaining datasets were used as training and validation datasets in a ratio of 90:10, respectively. Data augmentation was performed on the training dataset to increase the number and variation of photographs.

Augmentation includes random rotation, random vertical flip, and random horizontal flip. The angle of random rotation ranged from −45 to 45 degrees. The augmentation was performed using the ImageDataGenerator function in the Tensorflow library. The function will generate batches of tensor image data with real-time data augmentation.

Usually, each pixel of an 8-bit JPEG image will have a value ranging from 0 to 255, which is not appropriate for use with a CNN. Therefore, all photographs were further normalized by dividing each value by 255, so that their pixel values ranged from 0 to 1. Finally, each photograph was resized to a fixed dimension of 256 × 256 pixels using the bilinear interpolation method.

This research was approved by the Ethics Review Board of Rangsit University (DPE. No. RSUERB2022–064).

### CNN for OLP and Non-OLP Diagnosis

CNN is one of the neural network models in deep neural networks which is most applied to analyzing visual imagery. There are other neural network models that perform well on different types of data as well, such as fully connected neural network, or recurrent neural network.

The widely-used CNN models include AlexNet, VGG-16, Xception, and ResNet-50. In this study, the Xception, ResNet152V2, and EfficientNetB3 models were chosen for OLP and non-OLP lesion diagnosis because of their low model complexity and high classification accuracy on the ImageNet classification 19 20 21 task summarized by Keras. The difference between each neural network is the architecture of the neural network such as the number of parameters, number of layers (i.e., depth), or the computation time as shown in Table 1 . The number of layers reported are all layer that contains tunable parameters (i.e., changed over time due to the training process). The types and order of the layer in each neural network are also different that had already been explained in previous study. 19 20 21

**Table 1:** Accuracy, number of parameters, number of layers, and inference time of the selected model evaluated using Tesla A100 GPU when classifying ImageNet dataset

Model	Accuracy	Parameters	Number of layers	Time
**Xception** 19	79.0%	22.9M	81	8.1 ms
**ResNet152V2** 20	78.0%	60.4M	107	6.6 ms
**EfficientNetB3** 21	81.6%	12.3M	210	8.8 ms

**Fig. 1:**
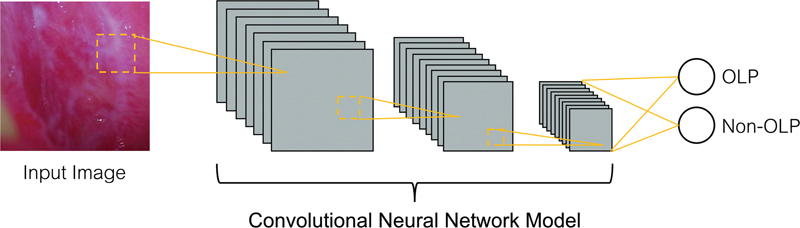
Framework of the method for oral lichen planus (OLP) and non-OLP lesion diagnosis using convolutional neural network.

The weights of the models were randomly generated and optimized using adaptive moment estimation 22 with a categorical cross-entropy loss, where the learning rate for updating the weight is fixed at 0.001. A batch size of 64 was set using a 100-epochs training process. The model was trained to classify two classes of data (OLP and non-OLP), as previously mentioned and shown in Fig. 1 .

**Fig. 2:**
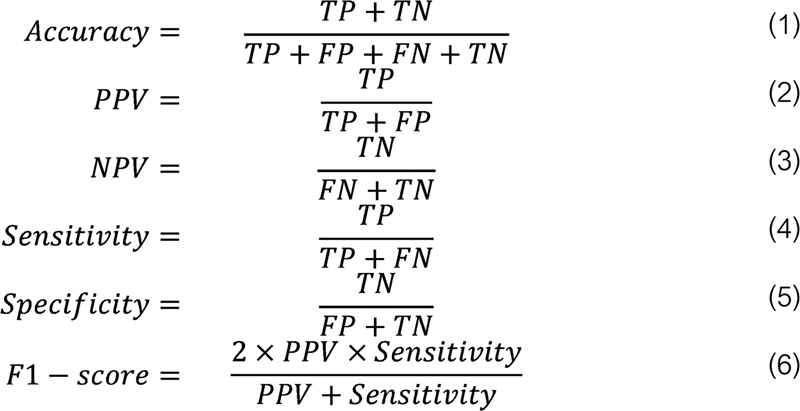
Metrics of performance for CNN models in OLP and non-OLP diagnosis. CNN, convolutional neural network; FN, false negative; FP, false positive; NPV, negative predictive value; OLP, oral lichen planus; PPV, positive predictive value; TN, true negative; TP, true positive.

Training was performed on a workstation with 1× NVIDIA GeForce RTX 3090 Ti graphics processing unit (24 GB memory). The program was developed with the relevant algorithms from Python3.6 and TensorFlow2.4 on an Ubuntu platform. The performance of the model was evaluated at every epoch during the training using the inference loss and accuracy from the validation dataset. After the training was completed, the weights of the epoch with the lowest validation loss were used to diagnose each photograph in the testing dataset. Performance metrics for the CNN model performance in OLP and non-OLP diagnosis included accuracy, positive predictive value (PPV) (i.e., precision), negative predictive value (NPV), sensitivity (i.e., recall), specificity, and F1-score. The equation for each metric is summarized in Fig. 2 . A true positive (TP) implies that the photograph is an OLP and the model also predicts that it is an OLP. A false negative (FN) indicates that the photograph is an OLP but the model predicts a non-OLP. A false positive (FP) indicates that the photograph is a non-OLP, but the model predicts an OLP. A true negative (TN) indicates that the photograph is a non-OLP and the model also predicted a non-OLP. Accuracy reflects the overall performance of the model. PPV and NPV represent the proportion of correctly diagnosed photographs among the total photographs retrieved by the model in its own class. Both sensitivity and specificity focus on the proportion of correct predictions. While sensitivity measures the proportion of correctly predicted positives out of all the actual positive values, specificity measures the proportion of correctly predicted negatives out of all the actual negative values. The F1-score is the harmonic average of PPV and sensitivity, which reflects the robustness of the model.

### Model Visualization and Case Review

Gradient-weighted class activation mapping (Grad-CAM) 23 uses the gradients flowing into the last convolutional layer to create a map that localizes and highlights important regions relevant to model prediction in an image. In this study, Grad-CAM was also used to visualize the important regions associated with discriminative clinical features on which the model relies. The red area indicates the more important features, whereas the blue color indicates the opposite. This color visualization reveals the underlying mechanism of the model's prediction. The photograph with the red color visualized in an unusual area was further analyzed by an experienced pathologist by reviewing the photograph along with the model visualization to determine the potential causes of such a scenario.

## Results

**Fig. 3:**
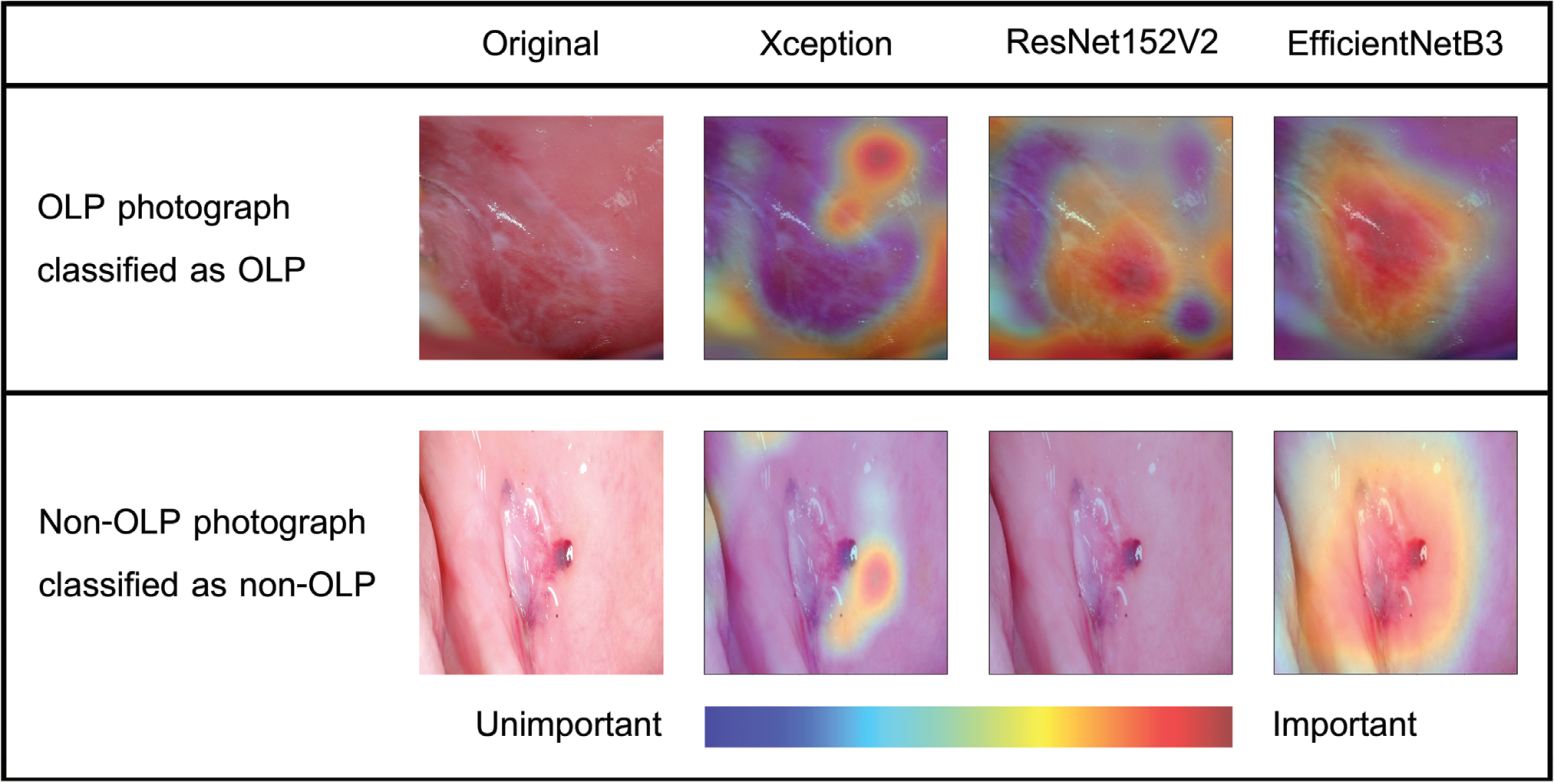
Gradient-weighted class activation mapping visualization of convolutional neural network classification for oral lichen planus (OLP) and non-OLP lesions (traumatic ulcer) from Xception, ResNet152V2, and EfficientNetB3 models.

The accuracy, number of parameters, and inference time of the selected model evaluated using Tesla A100 GPU when classifying ImageNet dataset were shown in Table 1 . Grad-CAM demonstrating the identified region of an OLP or a non-OLP lesion is shown in Fig. 3 . The red area indicates the more important features, whereas the blue color indicates the opposite. The performances of the three CNNs for OLP and non-OLP diagnoses on the test dataset after training using the same training parameters are summarized in Table 2 and Table 3 .

**Table 2:** Confusion matrix of each model in predicting OLP and non-OLP lesions using the photographs in the test dataset

Model		Actual OLP	Actual non-OLP
Xception	Predicted OLP	51 (TP)	9 (FN)
	Predicted non-OLP	4 (FP)	46 (TN)
ResNet152V2	Predicted OLP	42 (TP)	4 (FN)
	Predicted non-OLP	13 (FP)	51 (TN)
EfficientNetB3	Predicted OLP	53 (TP)	18 (FN)
	Predicted non-OLP	2 (FP)	37 (TN)

Abbreviations: FN, false negative; FP, false positive; OLP, oral lichen planus; TN, true negative; TP, true positive.

**Table 3:** Performance of each model in diagnosing OLP and non-OLP lesions using the photographs in the test dataset

Model	Accuracy	PPV	NPV	Sensitivity	Specificity	F1-score
Xception	88.18%	85.00%	92.00%	92.73%	83.64%	88.70%
ResNet152 V2	84.55%	91.30%	79.69%	76.36%	92.73%	83.17%
EfficientNetB3	81.82%	74.65%	94.87%	96.36%	67.27%	84.13%

Abbreviations: FN, false negative; FP, false positive; NPV, negative predictive value; OLP, oral lichen planus; PPV, positive predictive value; TN, true negative; TP, true positive.

Table 2 shows the prediction results in the confusion matrix and Table 3 presents the overall accuracy, PPV, NPV, sensitivity, specificity, and F1-score derived from Table 2 .

**Fig. 4:**
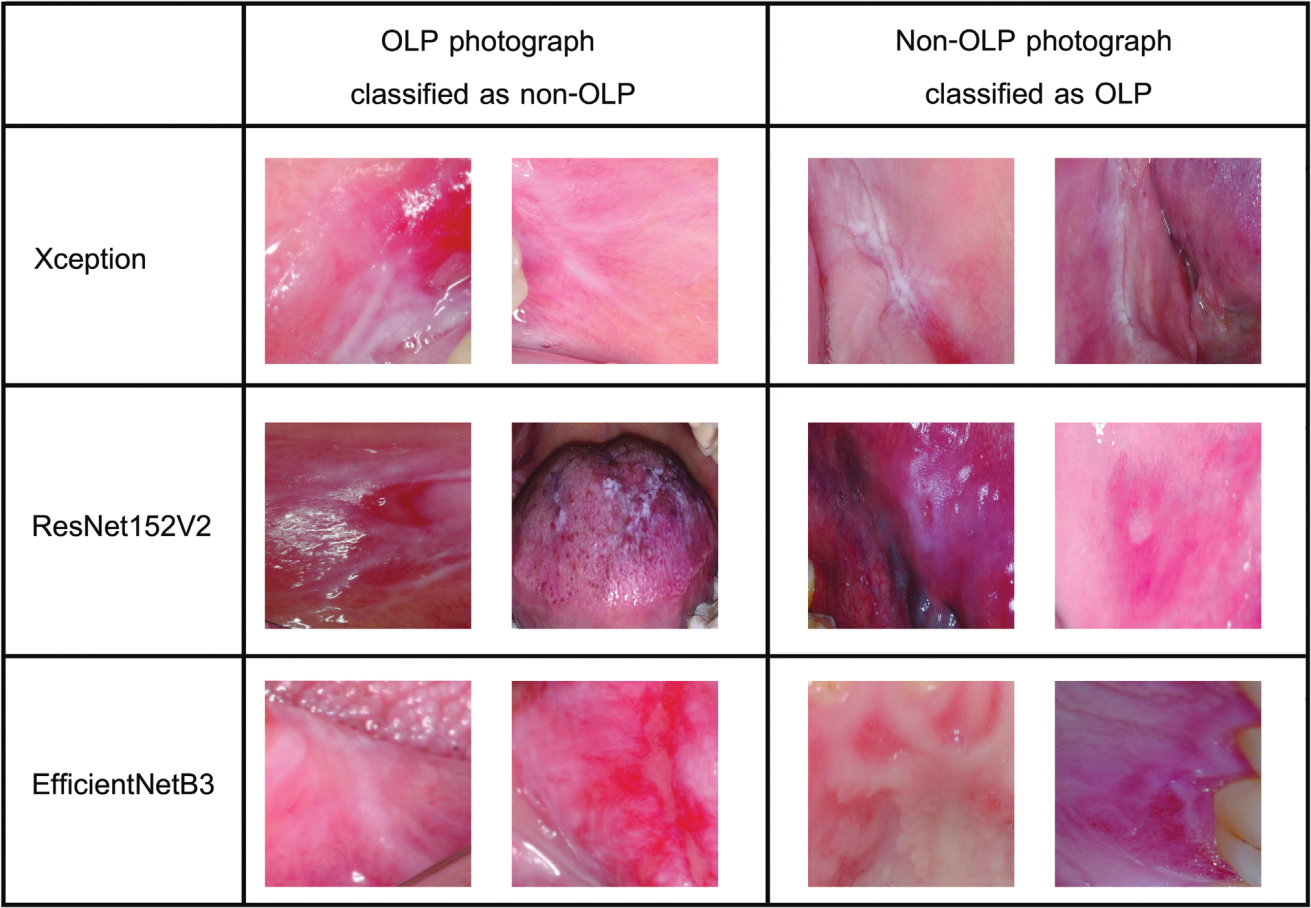
Misclassification photographs for Xception (hyperkeratosis), ResNet152V2 (traumatic ulcer), and EfficientNetB3 models (lupus erythematosus). OLP, oral lichen planus.

In Table 2 , the bold font denotes the number of photographs that each model correctly diagnosed. The table 2 shows that most of the OLP and non-OLP photographs were correctly diagnosed. The Xception and ResNet152V2 models performed well on both OLP and non-OLP photographs. However, the EfficientNetB3 model worked best on OLP photographs only. The potential causes of misclassification will be discussed later. The misclassification photographs of OLP and non-OLP were shown in Fig. 4 . Non-OLP misclassification cases for Xception model are three cases of traumatic ulcer, two cases of epithelial dysplasia, and one case of carcinoma in situ, recurrent aphthous ulcer, lupus erythematosus, and hyperkeratosis. Non-OLP misclassification cases for ResNet152V2 model are three cases of traumatic ulcer and one case of recurrent aphthous ulcer, while in EfficientNetB3 are seven cases of erythematous candidiasis, seven cases of traumatic ulcer, two cases of recurrent aphthous ulcer, and one case of hyperkeratosis and lupus erythematosus.

In Table 3 , the bold font denotes the best performance for a particular measurement included in the investigation. From this table, it is evident that all the selected CNN models were able to diagnose OLP and non-OLP lesions using photographs. The performance of the Xception model was significantly higher than that of the other models in terms of overall accuracy and F1-score of 88 and 89%, respectively. For instance, the overall accuracy of the Xception model was up to 6% higher than that of the other models. Even though the sensitivity and specificity scores of the Xception model may be lower than those of the other models, the overall model performance can still be considered satisfactory with such a limited dataset.

## Discussion

Clinical diagnosis by human experts was considered the gold standard until the development of AI systems using CNN showed superior results. 24 AI techniques using CNN for disease diagnosis (classification) have been studied in many fields using radiography, clinical examination, or histopathology. 7 8 9 10 11 12 13 14 15 16 17 18 The application of CNN in diagnosing skin lesions based on the clinical appearance and color has been studied previously. 25 26 Skin and oral mucosal lesions share similar diagnostic principles. The difference in color, such as white, red, white-red, brown-black, and yellow and changes in texture, such as ulcerated and vesiculobullous are the criteria for diagnosing oral lesions. The classic presentation of OLP is a white reticular lesion. Erosive, atrophic, bullous, and plaque lesions are the only accepted subtypes of OLP in the presence of reticular lesions elsewhere in the oral mucosa. 1 The application of CNN in OLP diagnosis has not been published previously. The classification model using CNN devised in this study is the first to use CNN algorithms for the diagnosis of OLP from photographs. OLP is considered an OPMD with a malignant transformation rate ranging from 0.5 to 2.28%. 5 27 28 Therefore, early diagnosis of OLP, which would lead to prompt treatment and prevention of oral cancer, is very important. Topical steroids are the treatment of choice for OLP. In recalcitrant severe lesions, systemic steroids, immunosuppressive agents, and biological agents are helpful. 2 Delayed diagnosis of OLP results in a poor quality of life because of the painful and extensive untreated lesion.

The variety of clinical manifestations of OLP makes its clinical diagnosis difficult for general practitioners who lack experience in diagnosing soft tissue lesions. The differential diagnoses of OLP include oral leukoplakia, hyperplastic candidiasis, oral ulcers, and other autoimmune mucocutaneous disorders, such as pemphigus vulgaris, oral pemphigoid, and lupus erythematosus. A definitive diagnosis of OLP can be made through histopathological diagnosis and immunofluorescence studies. Autofluorescence and chemiluminescence have been applied to increase the specificity and sensitivity of diagnostic methods in identifying white, red, and ulcerated lesions while diagnosing oral cancer or OPMD. However, these techniques show varying results and are subjective, depending on the experience with the device. 29 The correct diagnosis of OLP is crucial because the treatment varies for different lesions.

The development of telemedicine and teledentistry will benefit from the CNN models. The application of the CNN system with a smartphone may be helpful in teledentistry or may be used to screen lesions before consultation or referral to a specialist. Using both AI technology and CNN as diagnostic aids with careful clinical examination and history taking may be used as a diagnostic tool for disease diagnosis in the future.

In this study, we developed a new dataset that can be used to train and develop ML models for diagnosing OLP and non-OLP lesions using clinical photographs. The demonstration shows that CNN models can achieve an accuracy of 82 to 88% on a very small dataset, which is consistent with several previous studies that demonstrated the superiority of image augmentation for small datasets. 16 30 Among the three models, the Xception model performed the best in terms of both accuracy and F1-score. The Xception model was designed in 2017 to provide higher accuracy than previous CNNs, including ResNet152V2, in the ImageNet data classification task. The modification was motivated by the inception module in Inception-v3, which uses a modified depthwise separable convolutional layer (i.e., pointwise convolution followed by depthwise convolution). Therefore, it is understandable that the Xception model may achieve a better accuracy than ResNet152V2 in other classification tasks.

Subsequently, EfficientNetB3 was designed in 2019 to outperform the other CNNs in the ImageNet data classification task. It provides even higher accuracy than the Xception model in the ImageNet data classification task (see Table 1 ). However, in our study, it was interesting to note that EfficientNetB3 performed the worst in OLP and non-OLP diagnosis. This could be because EfficientNetB3 was created by performing a neural architecture search using the AutoML MNAS framework (i.e., automatic CNN model design framework). 31 The AutoML framework includes a process that uses the classification accuracy of the provided dataset to improve the structure of the model occasionally. Because the original target of the EfficientNetB3 model was to classify ImageNet data, the model was specially designed for the ImageNet dataset. Therefore, EfficientNetB3 may not be suitable for use with a classification task that is completely different from the ImageNet dataset.

The sensitivity for the detection of OLP from this model is comparable with that of other studies involving dental caries (81.90%). 8 The application results of CNN for the diagnosis of odontogenic cysts using panoramic and cone beam computed tomography or cyst and tumor models are also comparable with those of our study. 17 32 However, our result may be lower when compared with CNN-assisted oral cancer diagnosis because oral cancer is clinically easier to diagnose than OLP. 13 33 34 The incidence of oral cancer is higher than that of OLP; therefore, the data acquired for analysis are much easier to obtain. Most non-OLP misclassification are traumatic ulcer, recurrent aphthous ulcer and erythematous candidiasis. This is quite interesting because these groups of lesions are easier to diagnose than other lesions including in the differential diagnoses list for OLP such as epithelial dysplasia, pemphigus vulgaris, mucous membrane pemphigoid which AI performs well. This result emphasizes that AI may be helpful in differential diagnose of these difficult lesions for general practitioner

This study has some limitations. First, we included limited data in this study. Collecting more data from multiple centers would improve the sensitivity and F1-score of the models. Another limitation is that we have not used deep neural network to distinguish OLP lesions from oral lichenoid drug reactions, oral lichenoid contact lesions, or oral lichenoid of graft-versus-host diseases, because of their marked similarity in not only the clinical appearances but also the histopathologic features and characteristics upon fluorescence examination. 35 Clinical history taking is important for the diagnosis of these OLP subgroups. If more cases of different OLP subtypes can be accumulated, it would be interesting to determine whether CNN can classify these lesions.

In summary, the use of CNN to differentiate between OLP and non-OLP lesions yields favorable results. This result can be applied and expanded to the diagnosis of other oral lesions, such as white lesions, oral ulcers, or immune-induced oral lesions. These benefits can be applied to teledentistry, or the model may be transferred to a smart mobile application for easy use.

## References

[1] van der Meij E H van der Waal I Lack of clinicopathologic correlation in the diagnosis of oral lichen planus based on the presently available diagnostic criteria and suggestions for modifications J Oral Pathol Med 2003 32 09 507 512 12969224 10.1034/j.1600-0714.2003.00125.x

[2] Lodi G Manfredi M Mercadante V Murphy R Carrozzo M Interventions for treating oral lichen planus: corticosteroid therapies Cochrane Database Syst Rev 2020 2 02 CD001168 10.1002/14651858.CD001168.pub3 32108333 PMC7047223

[3] Oliveira Alves M G Almeida J D Balducci I Guimarães Cabral L A Oral lichen planus: a retrospective study of 110 Brazilian patients BMC Res Notes 2010 3 03 157 10.1186/1756-0500-3-157 20525297 PMC2898663

[4] Warnakulasuriya S Kujan O Aguirre-Urizar J M Oral potentially malignant disorders: a consensus report from an international seminar on nomenclature and classification, convened by the WHO Collaborating Centre for Oral Cancer Oral Dis 2021 27 08 1862 1880 33128420 10.1111/odi.13704

[5] Giuliani M Troiano G Cordaro M Rate of malignant transformation of oral lichen planus: a systematic review Oral Dis 2019 25 03 693 709 29738106 10.1111/odi.12885

[6] Carrozzo M Carbone M Gandolfo S Valente G Colombatto P Ghisetti V An atypical verrucous carcinoma of the tongue arising in a patient with oral lichen planus associated with hepatitis C virus infection Oral Oncol 1997 33 03 220 225 9307733 10.1016/s0964-1955(96)00073-5

[7] Alabi R O Bello I O Youssef O Elmusrati M Mäkitie A A Almangush A Utilizing deep machine learning for prognostication of oral squamous cell carcinoma-a systematic review Front Oral Health 2021 2 686863 35048032 10.3389/froh.2021.686863 PMC8757862

[8] Zhang X Liang Y Li W Development and evaluation of deep learning for screening dental caries from oral photographs Oral Dis 2022 28 01 173 181 33244805 10.1111/odi.13735

[9] Rao R S Shivanna D B Mahadevpur K S Deep learning-based microscopic diagnosis of odontogenic keratocysts and non-keratocysts in haematoxylin and eosin-stained incisional biopsies Diagnostics (Basel) 2021 11 12 2184 10.3390/diagnostics11122184 34943424 PMC8700488

[10] Ariji Y Fukuda M Nozawa M Automatic detection of cervical lymph nodes in patients with oral squamous cell carcinoma using a deep learning technique: a preliminary study Oral Radiol 2021 37 02 290 296 32506212 10.1007/s11282-020-00449-8

[11] Fu Q Chen Y Li Z A deep learning algorithm for detection of oral cavity squamous cell carcinoma from photographic images: a retrospective study EClinicalMedicine 2020 27 100558 10.1016/j.eclinm.2020.100558 33150326 PMC7599313

[12] Shavlokhova V Sandhu S Flechtenmacher C Deep learning on oral squamous cell carcinoma ex vivo fluorescent confocal microscopy data: a feasibility study J Clin Med 2021 10 22 5326 10.3390/jcm10225326 34830608 PMC8618824

[13] Yang S Y Li S H Liu J L Histopathology-based diagnosis of oral squamous cell carcinoma using deep learning J Dent Res 2022 101 11 1321 1327 35446176 10.1177/00220345221089858

[14] Ariji Y Yanashita Y Kutsuna S Automatic detection and classification of radiolucent lesions in the mandible on panoramic radiographs using a deep learning object detection technique Oral Surg Oral Med Oral Pathol Oral Radiol 2019 128 04 424 430 31320299 10.1016/j.oooo.2019.05.014

[15] Ahn Y Hwang J J Jung Y H Jeong T Shin J Automated mesiodens classification system using deep learning on panoramic radiographs of children Diagnostics (Basel) 2021 11 08 1477 34441411 10.3390/diagnostics11081477 PMC8394484

[16] Duman S Yılmaz E F Eşer G Detecting the presence of taurodont teeth on panoramic radiographs using a deep learning-based convolutional neural network algorithm Oral Radiol 2023 39 01 207 214 35612677 10.1007/s11282-022-00622-1

[17] Lee J H Kim D H Jeong S N Diagnosis of cystic lesions using panoramic and cone beam computed tomographic images based on deep learning neural network Oral Dis 2020 26 01 152 158 31677205 10.1111/odi.13223

[18] Son D M Yoon Y A Kwon H J An C H Lee S H Automatic detection of mandibular fractures in panoramic radiographs using deep learning Diagnostics (Basel) 2021 11 06 933 10.3390/diagnostics11060933 34067462 PMC8224557

[19] Xception F C Deep learning with depthwise separable convolutionsIEEE Conference on Computer Vision and Pattern Recognition (CVPR).2017 1800 1807 10.1109/CVPR.2017.195

[20] He Z X Ren K S Sun J Identity mappings in deep residual networksEuropean conference on computer vision. 2016: 630–645. Accessed December 6, 2022, at:https://arxiv.org/abs/1603.05027

[21] Quoc V L Tan M Efficientnet: Rethinking model scaling for convolutional neural networks International Conference on Machine Learning. Long Beach, California, PMLR. 2019:6105–6114. doi: */10.48550/arXiv.1905.11946*

[22] Ba JDP Kingma. Adam: A method for stochastic optimization. ICLR 2014. Accessed December 6, 2022 at:https://arxiv.org/pdf/1412.6980.pdf

[23] Cogswell M Selvaraju R R Das A Vedantam R Parikh D Batra D Grad-cam: Visual explanations from deep networks via gradient-based localizationProceedings of the IEEE International Conference on Computer Vision.2017 618 626

[24] Esteva A Kuprel B Novoa R A Dermatologist-level classification of skin cancer with deep neural networks Nature 2017 542(7639):115 118 28117445 10.1038/nature21056 PMC8382232

[25] Lyakhov P A Lyakhova U A Nagornov N N System for the recognizing of pigmented skin lesions with fusion and analysis of heterogeneous data based on a multimodal neural network Cancers (Basel) 2022 14 07 1819 10.3390/cancers14071819 35406591 PMC8997449

[26] Ramadan R Aly S Abdel-Atty M Color-invariant skin lesion semantic segmentation based on modified U-Net deep convolutional neural network Health Inf Sci Syst 2022 10 01 17 10.1007/s13755-022-00185-9 35978865 PMC9376187

[27] González-Moles M A Ramos-García P Warnakulasuriya S An appraisal of highest quality studies reporting malignant transformation of oral lichen planus based on a systematic review Oral Dis 2021 27 08 1908 1918 33274561 10.1111/odi.13741

[28] Fitzpatrick S G Hirsch S A Gordon S C The malignant transformation of oral lichen planus and oral lichenoid lesions: a systematic review J Am Dent Assoc 2014 145 01 45 56 24379329 10.14219/jada.2013.10

[29] Huber M A Adjunctive diagnostic techniques for oral and oropharyngeal cancer discovery Dent Clin North Am 2018 62 01 59 75 29126494 10.1016/j.cden.2017.08.004

[30] Figueroa K C Song B Sunny S Interpretable deep learning approach for oral cancer classification using guided attention inference network J Biomed Opt 2022 27 01 15001 10.1117/1.JBO.27.1.015001 PMC8754153 35023333

[31] Chen B Tan M Pang R Mnasnet: Platform- aware neural architecture search for mobileProceedings of the IEEE/CVF Conference on Computer Vision and Pattern Recognition;2019 2820 2828

[32] Yang H Jo E Kim H J Deep learning for automated detection of cyst and tumors of the jaw in panoramic radiographs J Clin Med 2020 9 06 E1839 10.3390/jcm9061839 PMC7356620 32545602

[33] Warin K Limprasert W Suebnukarn S Jinaporntham S Jantana P Automatic classification and detection of oral cancer in photographic images using deep learning algorithms J Oral Pathol Med 2021 50 09 911 918 34358372 10.1111/jop.13227

[34] Lin H Chen H Weng L Shao J Lin J Automatic detection of oral cancer in smartphone-based images using deep learning for early diagnosis J Biomed Opt 2021 26 08 86007 10.1117/1.JBO.26.8.086007 PMC8397787 34453419

[35] Lodolo M Gobbo M Bussani R Histopathology of oral lichen planus and oral lichenoid lesions: An exploratory cross-sectional study Oral Dis 2023 29 03 1259 1268 34951080 10.1111/odi.14112

